# The Effects of Dietary Nutrition on Sleep and Sleep Disorders

**DOI:** 10.1155/2020/3142874

**Published:** 2020-06-25

**Authors:** Mingxia Zhao, Houzhen Tuo, Shuhui Wang, Lin Zhao

**Affiliations:** ^1^Department of Neurology, Beijing Friendship Hospital, Capital Medical University, Beijing, China; ^2^Department of Cardiology, Beijing Anzhen Hospital, Capital Medical University, Beijing, China

## Abstract

Sleep disorder significantly affects the life quality of a large number of people but is still an underrecognized disease. Dietary nutrition is believed to play a significant impact on sleeping wellness. Many nutritional supplements have been used trying to benefit sleep wellness. However, the relationship between nutritional components and sleep is complicated. Nutritional factors vary dramatically with different diet patterns and depend significantly on the digestive and metabiotic functions of each individual. Moreover, nutrition can profoundly affect the hormones and inflammation status which directly or indirectly contribute to insomnia. In this review, we summarized the role of major nutritional factors, carbohydrates, lipids, amino acids, and vitamins on sleep and sleep disorders and discussed the potential mechanisms.

## 1. Introduction

The body maintains a biological rhythm called circadian rhythm which oscillate in cycles of 24 hours. This normal circadian rhythm orchestrates normal physiological cycles happening each day [1]. Sleep disorder is a highly prevalent disease that disrupts the normal circadian rhythm which negatively impacts psychological well-being and physical health [2]. There are several types of sleep disorders, among which insomnia, obstructive sleep apnoea (OSA), and circadian rhythm disorders are more frequently studied [3]. Sleep disorders are not only associated with decreased life quality and work efficiency but also with increased medical and psychiatric problems [4]. It is considered a risk factor for many diseases including cardiovascular events [5, 6], hypertension [7, 8], and type 2 diabetes [9]. Sleep wellness of children is associated with their physiological and mental health as well as cognition development and behaviors [10].

The circadian rhythm is controlled by both the internal genetic components of biological clock (clock genes) and external factors including those from nutrition and environment. Diet is believed to play an important role in the regulation of sleep wellness [11]. The mechanism for diet in the regulation of sleep is a very complex question that could be demonstrated through the following pathways. First, diet components can directly affect sleep. For example, caffeine which is contained in caffeinated coffee or tea causes a decrease in total sleep time and quality, as well as an increase in sleep induction time [12]. Caffeine is chemically related to adenosine, which is a sleep-inducing agent. It is believed that caffeine works by reversibly antagonizing the sleep-inducing adenosine receptors (A_2A_R) in the brain, although other pathways may coexist [13]. Melatonin is a well-recognized sleep inducer which conveys information of the daily cycle of light and darkness to the body. Melatonin activates two receptors, MT1 and MT2, both are G-protein-coupled receptors to mediate its effects on sleep induction and circadian rhythm. Thus, food containing melatonin can directly have an effect on sleep [14]. Secondly, many nutritional metabolites can be bioactive in sleep regulation directly or through the regulation of other relating factors as discussed below. It should be noted that nutrition could significantly alter the commensal microbiota which could affect the metabolic generation of metabolites [15]. Thirdly, long-term nutritional factors could alter the inflammation status which is also closely related with insomnia. This has been supported by substantial number of studies that sleep disturbance is related with altered circulating inflammatory cytokines (especially C-reactive protein and interleukine 6) and glucocorticoids [16–20]. The relationship of diet patterns and inflammation status has been reviewed previously [21], thus is no longer a focus of current review. It is worth to mention that with the establishment of the link between chronic inflammation and many major diseases of modern society, this area is receiving more and more research interests. However, the mechanism of inflammation on sleep wellness is still a complex question which requires more investigation.

The past decades have seen a dramatic increase in literature on the role of diet/nutrition on sleep. However, although huge in quantity, many of which sleep are observational with a limited sample size and often had contradictory results. This makes those studies based on clinical interventions more valuable to unveil the role of each nutritional components. Moreover, due to the complex nutritional component of food, studies involving refined nutrition components provided better insights to that particular nutrition. Thus, this review summarized the knowledge on the nutritional components on sleep with more preferential focus on those studies with clinical interventions and refined nutrition.

## 2. Carbohydrates

Instead of studying each refined carbohydrate, the overall dietary glycemic index (GI) based on their effects on postprandial blood glucose levels is normally used to study the impact of carbohydrate on diseases [22]. High-GI diet has been shown to be associated with stroke [23], cancer [24], and certain chronic diseases [25]. The consumption of high-GI food caused a rapid increase in blood glucose level which results in compensatory insulin increase and a series of downstream humoral effects. The studies on the role of carbohydrates on sleep have mixed results. Afaghi et al. reported that healthy sleepers (12 healthy adults ranging from 18-35 years old) taking carbohydrate-based high-GI meal 4 h before bedtime resulted in a significant shortening of sleep onset latency (SOL, 48.6% reduction) when compared to individuals taking a low-GI meal [26]. The result is supported by another study that low-carbohydrate intake was associated with difficulty maintaining sleep [27]. However, other studies, including a recently published by Gangwisch et al., suggested that high glycemic index and glycemic load diets is a risk factor for insomnia [28]. This prospective study of a much larger population of postmenopausal women population demonstrated that high-GI diet was associated with increased insomnia incidence over 3 years, and higher intakes of dietary added sugars, starch, and nonwhole/refined grains each were associated with higher incidence of insomnia. Moreover, they found higher fiber content in food as well as nonjuice fruit were associated with a lower prevalence and incidence of insomnia. This conclusion is consistent with a previous study that high intake of confectionary is related with poor sleep quality among middle-aged female Japanese workers [29]. Supporting this concept, a study on short-term consumption of a very low-carbohydrate (VLC) diet over 48 h comparing to a control mixed diet on sleep indices suggested promotes SWS (deep sleep stage) and reduces the percentage of REM sleep (“dreaming” sleep) [30].

Although not completely solved, the potential mechanisms behind the relationship of carbohydrate and insomnia has been suggested. Food with high GI could alter the ratio of tryptophan relative to other large neutral amino acids (LNAAs including tyrosine, phenylalanine, leucine, isoleucine, valine, and methionine) in the circulation [31]. It does so through the effect of insulin which increased following consumption of high-GI food [31]. Insulin promotes the selective uptake of LNAAs by the muscles leading to higher tryptophan to LNAA ratio. Since tryptophan competes with LNAA for transportation into the brain [32], this change in ratio may lead to increased tryptophan in the brain [26]. Tryptophan is the precursor for serotonin which induces sleep. Brain serotonin levels could indeed increase after ingestion of carbohydrate [33]. This mechanism was used to explain the observations that high-GI diet benefits sleeping [26]. However, this theory has been challenged by the recent publication by Gangwisch et al. who suggested that this theory may not be realistic as it required the meal to contain only carbohydrate. If the meal contains as little as 5% protein, this can prevent the increase of tryptophan concentrations [28]. Moreover, the increase of serotonin is not necessarily associated with melatonin whose production is regulated by the presence of darkness [28]. Instead, they proposed that hyperglycemia induced after high-GI diet and resulting compensatory hyperinsulinemia could induce the release of autonomic counterregulatory hormones including adrenaline, cortisol, glucagon, and growth hormone which contributed to insomnia [28, 34]. Moreover, high-GI diets have also been shown to stimulate inflammatory immune responses [35] and lead to alternations in intestinal microbiome which may also profoundly affect sleep quality [15].

It should be noted that these abovementioned studies are performed in different populations with dramatically different sample sizes and experiment designs, so the results may be comparable to each other. Nevertheless, more studies are needed to address the relationship between high-carbohydrate diet and insomnia from a mechanistic aspect.

## 3. Fatty Acids

Fatty acids are another major component of human diet, including saturated fat and unsaturated fat [36]. High consumption of saturated fat increases low-density lipoprotein (LDL) cholesterol levels and is related with increased risks for diseases like cardiovascular diseases [37] and diabetes [38]. Among unsaturated fats, omega-3 polyunsaturated fatty acids (PUFAs) including *α*-linolenic acid (ALA), eicosapentaenoic acid (EPA), and docosahexaenoic acid (DHA) have been extensively studied on their effects to human health. Contrary to saturated fat, consumption of omega-3 PUFA is known to prevent the risks of cardiovascular diseases [39] and stroke [40]. The relationship between fatty acids and sleep wellness has also been studied and is reviewed [41].

### 3.1. Saturated Fatty Acids

Animal fat contains almost exclusively saturated fatty acids. Processed foods including those deep fried in hydrogenated oil are also high in saturated fat content. Consumption of saturated fat is a major risk factor for cardiovascular disease and diabetes as has been suggested by many scientific societies [42].

The studies on the role of saturated fatty acids on sleep are relatively rare. In a study of normal weighted adults, it is concluded that higher saturated fat intake during the day was associated with a shortened duration of slow wave sleep and more arousals during the night [43]. Another study of 459 postmenopausal women investigated the relationships among nutrients in the diet and objective sleep. The authors concluded that total sleep time as measured by actigraphy was negatively associated with intake of total fat and saturated fat [44]. From these limited studies, it seems that the consumption of saturated fatty acids deteriorates sleeping wellness. This is also true if diabetes is induced due to the long-term consumption of saturated fatty acids, as diabetes is often associated with sleeping problems [45].

### 3.2. Omega-3 PUFA

Omega-3 PUFA is a type of polyunsaturated fatty acid with good reputation for health. Compared to animal fats which are largely saturated, fish and vegetable contain significant portion of unsaturated fat. Omega-3 fats are important for the development of the brain. The deficiency in DHA in the developing brain will lead to problems in neurogenesis, associated with altered learning and visual problems [46]. In addition, omega-3 fats are considered anti-inflammatory, consumption of which can reduce the inflammation in the body that benefit a number of chronic diseases [47]; thus, omega-3 fats are commonly used as nutritional supplements to prevent cardiovascular problems and stroke.

Studies have suggested that diet deficient in omega-3 PUFA disturbed nocturnal sleep though affecting the melatonin rhythm and circadian clock functions [48]. There is also a positive relation between omega-3 fatty acid composition in gluteal adipose tissue and sleep wellness including slow wave sleep and rapid eye movement sleep among obese patients with obstructive sleep apnoea syndrome [49]. A study of healthy children has reported that higher blood DHA level is associated with significantly improved sleep wellness [50]. In their subsequent randomized controlled trial (RCT) of DHA supplementation (with 600 mg/day for 16 weeks), significant group differences were observed including sleep duration increased by 58 min and fewer and shorter night-wakings in the treatment group versus the placebo group [50]. Other than children, the effect of DHA on sleep was also reported in adolescents, as higher plasma DHA was associated with earlier sleep timing and longer weekend sleep [51].

Although the prevailing results suggested the beneficial role of omega-3 PUFA on sleep, a report raised opposite findings stating high-EPA fish oil supplements is likely associated with disturbance of sleeping after successful treatment of depression; the symptoms disappeared after cessation of supplementation [52]. However, such negative reports on omega-3 fat are rare. Although fish is a source of omega-3 fat, the results are mixed when it comes to the impact of fish consumption to sleeping wellness. A positive correlation is found between better sleep quality and oily fish consumption in a population of over 40 years old [53]. Moreover, a study of 95 male adults consuming Atlantic salmon three times per week from September to February gave a positive impact on sleep in general and also on daily functioning effect on resting HRV and EPA+DHA, but not on vitamin D status [54]. However, in a two-armed randomized controlled trial, there was no significant differences in mental health and sleep for the fish eating group compared with the meat eating group in kids of 4-6 years old [10].

### 3.3. Omega-6 PUFA

Omega-6 PUFA are another type of polyunsaturated fatty acid that is abundant in vegetable oil like corn, primrose seed, and soybean oil. Compared to the general consensus on the beneficial role of omega-3 fat on sleep, the roles of omega-6 are not as clear. Omega-6 fat serves as precursors of potent lipid mediators called eicosanoids. For example, arachidonic acid is the precursor for at least three groups of lipid mediators including, prostaglandins (PGs), thromboxanes, and leukotrienes [55]. Generally speaking, eicosanoids derived from omega-6 displayed a proinflammatory function while eicosanoids derived from omega-3 PUFA showed more anti-inflammatory tendency. The metabolism of omega-6 fatty acids and generation of eicosanoids as well as how they influence inflammatory responses have been reviewed [55].

The prostaglandin derivatives of arachidonic acid PGD_2_ and PGE_2_ are very important factors regulating sleep. PGD_2_ has been experimentally tested as an effective sleep promoter on different animal models [56–58]. This humoral factor is gradually accumulated in the brain while awake and circulates in the cerebrospinal fluid as a sleep hormone. In contrast to the sleep-inducing role of PGD_2_, PGE_2_ has a strong awakening effect in rats and suppresses sleep [59]. Considering the contrasting results of PGD_2_ and PGE_2_ on sleep induction, it would be interesting to know the results of increased omega-6 PUFA supplement, especially when arachidonic acid is provided. However, despite the well-established role of PGD_2_ and PGE_2_ in sleep regulation, studies on the consumption of their precursor omega-6 PUFA on sleep wellness are rare. In a bioinformatics study, lower arachidonic acid biosynthesis was seen in the insomnia group, suggesting that lower production of arachidonic acid may be associated with a high incidence of insomnia [60].

No studies directly supply omega-6 fatty acids to study their role on sleep. However, the ratio of omega-6 to omega-3 essential fatty acids (EFA) is commonly used to describe the fatty acid composition in the nutrition field. It is believed that a diet with omega-6/omega-3 ratio of approximately 1 is recommend, whereas this ratio has increased steadily over the past few decades (currently ~15 : 1). This imbalance is associated with many chronic inflammatory diseases such as nonalcoholic fatty liver disease, cardiovascular disease, obesity, inflammatory bowel disease (IBD), and rheumatoid arthritis. [55]. A 4-week double-blind study including 100 Alzheimer patients indicated supplement of compound comprising a 4 : 1 ratio of omega-6/omega-3 fatty acids improves sleep compared to placebo [61]. However, the mechanism of action is not clear; it is possible that the effect is indirect through the regulation of inflammation status.

## 4. Amino Acids

Amino acids are the building blocks of proteins. There are hundreds of naturally occurring amino acids and most of which can be found in the human diet. Numerous studies on the role of amino acids on sleep wellness and insomnia have been performed in the past decades. Current review only focuses on the most important amino acids in sleeping, including tryptophan, glutamine, tyrosine, and gamma-aminobutyric acid (GABA).

### 4.1. Tryptophan

Tryptophan is the substrate for serotonin which has been intensively studied on its role on sleep for many decades [20]. Although the role of serotonin on sleep has been under debate, there is a general agreement that serotonin is a major sleep mediator which first increases wakefulness but then increases NREM sleep [20]. Considering the role of serotonin, it has been indicated that supplementation of tryptophan (1 g or more) produces an increase in subjective sleepiness and a decreased time to sleep especially in subjects with mild insomnia [62]. A random double-blind experiment on healthy adults suggested that tryptophan consistently reduced sleep latency which is associated with blood levels [63]. Recently, a Japanese study of younger aged population concluded that tryptophan ingested during breakfast is required for children to keep a morning-type diurnal rhythm and maintain high quality sleep [64]; however, this study did not involve supplementation of tryptophan, instead they calculated the tryptophan-index based on food they consume.

### 4.2. Gamma-Aminobutyric Acid and Glutamine

Gamma-aminobutyric acid (GABA) is a bioactive amino acid with which does not form proteins. This amino acid has received significant research interests due to its effects on many metabolic disorders [65]. The production of GABA is through the decarboxylation of L-glutamate catalyzed by glutamate decarboxylase. Food fermented by lactic acid bacteria or yeast normally contains an increased level of GABA. Numerous physiological functions about GABA have been reported and reviewed in [65]. In particular, the sleep-promoting function of GABA has been appreciated [66].

There are many studies showing the sleep-promoting effect of GABA, for example, Byun et al. reported a study of 40 patients with insomnia receiving 4 weeks of GABA (300 mg/day) have decreased sleep latency and increased sleep efficacy [66]. The mechanisms of sleep induction by GABA through their receptors have been reviewed [67]. GABA receptor agonists have also been used to induce sleeping [68].

Glutamine is also a nonessential amino acid which can be used for the synthesis of GABA, a known inhibitory neurotransmitter and sleep inducer. Thus, it has been hypothesized and sometime taken for granted that the supplement of glutamine can benefit sleep. However, since glutamine is nonessential, this can be generated by the body. The beneficiary effects of glutamine supplementation, if exists, still need scientific confirmation.

### 4.3. Tyrosine

Tyrosine is a nonessential amino acid whose metabolite is norepinephrine (NE) which is a neurotransmitter. NE is released at its lowest levels during sleep and rises during wakefulness. The level of NE dramatically increases during situations of stress or danger, which is called the fight-or-flight response. NE is long known for its role in maintaining general arousal [69] which has also been confirmed using mouse models. Dopamine *β*-hydroxylase knockout mice, which lack norepinephrine displayed increased overall sleep and require stronger stimuli to wake up after sleep deprivation [70]. The precursor for NE dopamine (DA) also inhibits adrenergic receptor signaling and blocks the synthesis of melatonin through *α*1B-D4 and *β*1-D4 receptor heteromers [71]. The supplementation of tyrosine has been used in many cognitive/behavioral studies but yielding significantly varied results [72]. Magill et al. reported that supplementation of tyrosine 150 mg/kg following overnight sleep deprivation improved working memory, reasoning, and vigilance [73]. However, the role of tyrosine supplement on sleep disorders are not well studied. Considering the significant roles of tyrosine metabolites during sleep, it would be worthwhile to study this topic.

## 5. Vitamins

### 5.1. Vitamin D

Vitamin D is a fat-soluble vitamin which is crucial for the absorption of calcium and many other biological effects. The most important vitamin D3 and D2 can both be synthesized by the body in the presence of sunshine or obtained from the diet. Fatty fish is a major source of dietary vitamin D. Multiple studies have studied the role of vitamin D on sleep. A meta-analysis including 9 studies (6 cross-sectional, 2 case-control, and 1 cohort studies) aimed at clarifying the association between vitamin D and sleep disorder risk [74]. Overall, the study concluded that vitamin D deficiency is associated with a higher risk of sleep disorders including poor sleep quality, short sleep duration, and sleepiness [74]. When examining each individual studies, most studies indeed suggested positive correlation of vitamin D intake and sleep quality. Moreover, there is an association between serum vitamin D levels and obstructive sleep apnoea syndrome [75]. The mechanism regarding the role of vitamin D in sleep is yet to be confirmed, possibly related with inflammation and oxidative stress [75].

### 5.2. Vitamin C

Vitamin C found in most citrus fruits and vegetables has been shown to be protective of the brain against memory losses associated with sleep deprivation [76]. A study compared people with short sleep and those with longer sleep and concluded that the vitamin C are among those consumed less by short sleepers [77]. A cross-sectional study of adults in the UK suggested there is a relationship between fruit/vegetable intake and sleep wellness, and long sleepers have high plasma levels of vitamin C [78]. However, other than that, literature actually does not have much evidence supporting the relationship of vitamin C and sleep wellness.

### 5.3. Vitamin B6/B12

Vitamin B6 (pyridoxine) is widely distributed in food that serves as a coenzyme in hundreds of enzymatic reactions. A randomized, double-blind, placebo-controlled study of vitamin B6 and B vitamins on the effects on dreaming and sleep showed no significant differences in the B6-treated group compared with the placebo in terms of time awake during the night, sleep quality, or tiredness on waking. However, the B complex-treated group showed significantly lower self-rated sleep quality and significantly higher tiredness on waking. The authors suggested that vitamin B6 supplementation had no detrimental effects on sleep quality [79].

The effects of vitamin B12 on sleep is also controversial. A case report suggested successful vitamin B12 treatment for a free-running sleep-wake rhythm and delayed sleep phase syndrome [80]. However, a multicenter double-blind study challenged this conclusion showing that 3 mg vitamin B12 administered over 4 weeks is not effective for delayed sleep phase syndrome [81]. However, on animal models, intravenously administered vitamin B12 promotes effects on the sleep of rat, especially during the light period [82].

## 6. Concluding Remarks

It is easy to believe that dietary nutrition plays an important role in sleep wellness. Using diet management to improve sleep is a possible, convenient, and inexpensive strategy. Indeed, some nutritional components or their metabolites have been experimentally proved to be beneficial. However, many other are only hypothetical and lack solid scientific evidence. It is more complicated when it comes to the relationship of the consumption of a particular food and sleep wellness, due to complex composition of food, as well as the absorptive and metabolic abilities of each individual. Moreover, a majority of the studies are observational or cross-sectional, many of which included limited sample size and results from literature often conflict with each other. This field needs more high-quality cohort studies and randomized controlled trials (RCTs) to further confirm the contribution of dietary nutrition to sleep wellness. In addition, better animal models to mimic clinical situations are also of great importance.
